# A Neural Code That Is Isometric to Vocal Output and Correlates with Its Sensory Consequences

**DOI:** 10.1371/journal.pbio.2000317

**Published:** 2016-10-10

**Authors:** Alexei L. Vyssotski, Anna E. Stepien, Georg B. Keller, Richard H. R. Hahnloser

**Affiliations:** Institute of Neuroinformatics, Neuroscience Center Zurich, University of Zurich/ETH Zurich, Zurich, Switzerland; Boston University, United States

## Abstract

What cortical inputs are provided to motor control areas while they drive complex learned behaviors? We study this question in the nucleus interface of the nidopallium (NIf), which is required for normal birdsong production and provides the main source of auditory input to HVC, the driver of adult song. In juvenile and adult zebra finches, we find that spikes in NIf projection neurons precede vocalizations by several tens of milliseconds and are insensitive to distortions of auditory feedback. We identify a local isometry between NIf output and vocalizations: quasi-identical notes produced in different syllables are preceded by highly similar NIf spike patterns. NIf multiunit firing during song precedes responses in auditory cortical neurons by about 50 ms, revealing delayed congruence between NIf spiking and a neural representation of auditory feedback. Our findings suggest that NIf codes for imminent acoustic events within vocal performance.

## Author Summary

Transmission of birdsong across generations requires tight interactions between auditory and vocal systems. However, how these interactions take place is poorly understood. We studied neuronal activity in the brain area located at the intersection between auditory and song motor areas, which is known as the nucleus interface of the nidopallium. By recording during singing from neurons in the nucleus interface of the nidopallium that project to motor areas, we found that their spiking precedes peaks in vocal amplitudes by about 50 ms. Notably, quasi-identical notes produced at different times in the song motif were preceded by highly similar spike patterns in these projection neurons. Such local isometry between output from the nucleus interface of the nidopallium and vocalizations suggests that projection neurons in this brain area code for imminent acoustic events within vocal performance. In support of this conclusion, during singing, projection neurons do not respond to playback of white noise sound stimuli, and activity in the nucleus interface of the nidopallium precedes by about 50 ms neural activity in the avian analogue of auditory cortex. Therefore, we conclude that the role of neuronal activity in the nucleus interface of the nidopallium could be to link desired auditory targets to suitable motor commands required for hitting these targets.

## Introduction

Some highly skilled learned behaviors, such as speech or birdsong, are encoded primarily in dedicated cortical brain areas [1–4]. To assure integration with other behaviors and with the sensory environment, these cortical pattern generators must rely on inputs that convey information about motor plans, desired sensory targets, and sensory feedback [3,5,6]. However, the integration of motor signals with sensory, planning, and decision-related signals remains highly enigmatic.

Songbirds are ideally suited to decipher neural integration in skilled behaviors because they learn their courtship songs by hearing and memorizing a song template from a tutor and by adjusting their immature songs using auditory feedback [7–10]. Although calling in birds per se does not require cortical motor input [1], analogous to whisking in rodents [11], the highly stereotyped songs of adult birds are driven by the dedicated cortical area HVC [1,12], whose projection neurons fire ultra-sparsely in time [13,14].

Here we investigate HVC’s input from the cortical nucleus interface of the nidopallium (NIf). NIf is a thin plate of cells encapsulated between field L areas, from which NIf indirectly receives auditory input [15] and relays it to HVC [16–20]. NIf activity is required for song learning [6] as well as for normal song production [21,22], although song recovers within a few days after irreversible NIf lesions [23]. NIf neural activity increases during song production [24,25], but the motor and auditory-related firing patterns in NIf neurons that project to HVC (NIf_HVC_ neurons) have never been investigated in awake, singing birds.

We find that during song production, NIf neurons do not respond to auditory white noise stimuli, making it very unlikely that NIf relays auditory feedback to HVC. NIf_HVC_ spikes tend to precede song syllables, spike patterns are highly syllable-specific, and they occur in distinguished and stereotyped patterns prior to individual song notes. Highly similar notes in different song syllables are preceded by highly similar NIf_HVC_ spike patterns, revealing a locally isometric relationship between NIf output and vocal output. Multi-unit activity in NIf during singing is correlated with subsequent activity in field L, pointing at a neural code in NIf that anticipates auditory feedback.

## Results

In chronic recordings in freely moving animals, we identified NIf_HVC_ neurons by their antidromic responses to bipolar electrical stimulation in HVC (see Materials and Methods). Unless explicitly stated, the song-related NIf_HVC_ data are from a population of 20 neurons chronically recorded with motorized microdrives in six birds. In four separate birds, we recorded NIf and field L multiunit activity using 16-channel probes mounted to a manual microdrive.

### NIf_HVC_ Firing Rates Increase Prior to Song Onsets and Return to Baseline Prior to Song Offsets

**Fig 1:**
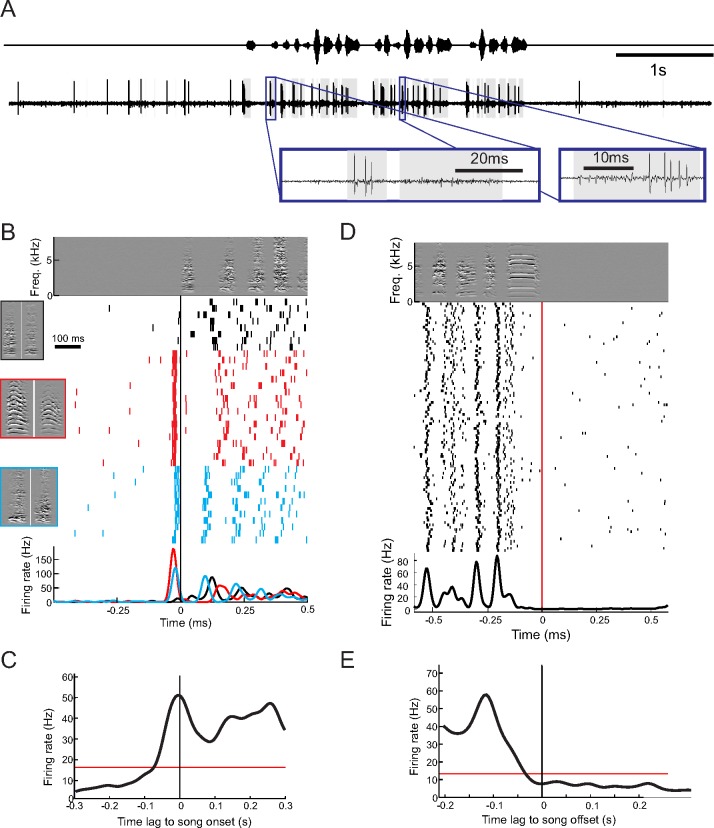
NIf_HVC_ firing rates increase prior to song onsets and return to baseline prior to song offsets. **A.** Song oscillogram (top) and raw extracellular trace of a NIf_HVC_ cell (bottom) depict baseline and song-related NIf_HVC_ firing. The insets show close-ups of spike bursts. The shaded gray area highlights time intervals during which the root-mean square (RMS) voltage exceeds a threshold of one standard deviation. **B.** Spike raster plot of a single NIf_HVC_ cell aligned with song onsets (example derivative sound spectrogram shown on top), revealing no anticipatory firing when the first syllable is a short call (black rasters), but distinctive bursts before songs starting with a long call (red rasters) or an introductory note (blue rasters). Example onset syllables are shown on the left, framed in corresponding colors. Average firing rate curves, in corresponding colors, are shown at the bottom. **C.** The onset-related firing rate averaged over *n* = 16 NIf_HVC_ cells (black full line) exceeds a threshold (red line) of three jackknife standard deviations above baseline, already firing 70 ms before song onset and peaking coincidently with song onset. **D.** Spike raster of same unit as in B, but aligned with song offset. The neuron fires at baseline rates already toward the middle of the last syllable. **E.** Aligned with song offsets, the average firing rate of *n* = 16 NIf_HVC_ cells falls below baseline threshold (three standard deviations, red), already 30 ms before song offset. All data can be downloaded as part of the supporting information files (S1 Data).

We found that NIf_HVC_ neurons were spontaneously active during quiescent non-singing periods and increased their firing rates before song onsets (Fig 1A–1C). In two juvenile birds that started their songs with either one of several types of introductory notes or calls, NIf_HVC_ neurons fired highly distinct spike patterns prior to these different onsets, ranging from almost no spikes prior to some note/call onsets, up to a high-frequency burst of more than four spikes prior to other onsets, revealing differential coding of song onset type.

On average, NIf_HVC_ firing rates gradually increased before song onset and significantly deviated from pre-song baseline firing (greater than 3 jackknife standard deviations) already 70 ms prior to song onset (Fig 1C). The firing rate during song of 37 Hz (averaged from -70 to +160 ms of song onsets) was much higher than the 8 Hz baseline firing rate (averaged from -300 to -100 ms before song onset, *p* = 0.0005 Wilcoxon signed rank test, *n* = 16 NIf_HVC_ cells, excluding four cells in which we did not record sufficient amounts of baseline activity prior to song onsets). NIf_HVC_ firing also returned to baseline prior to song offsets (Fig 1D). On average, NIf_HVC_ firing rates were indistinguishable from post-song baseline firing (less than three jackknife standard deviations, *n* = 16 cells) already 34 ms prior to song offset (Fig 1E). Thus, HVC receives excessive NIf input long before song onset, and this input wanes before song offset.

### NIf_HVC_ Spikes Precede Peaks in Sound Amplitude with Diverse Latencies across Cells and Syllables

**Fig 2:**
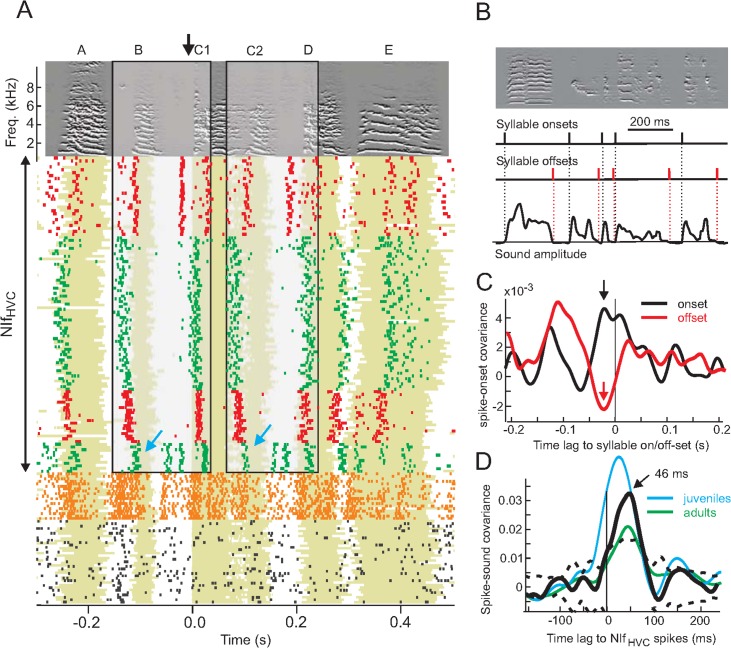
NIf_HVC_ cells fire stereotyped patterns of spikes during song motifs, mainly prior to syllable onsets. **A.** Spike raster plot in four antidromically identified NIf_HVC_ cells (red and green rasters, top) and two NIf interneurons (orange and black rasters, bottom), aligned with song-motifs (the black arrow indicates alignment point). Sound amplitudes (binarized, shaded green area) reveal jitter in song tempo, explaining increased variability of NIf_HVC_ spiking away from the alignment point. Syllable C looks like a concatenation of two other syllables (D and B). The concatenation is so tight that the bird seems to sing B-D-B-D with almost no gap in the transition from D to B (yet C1 and D, as well as C2 and B, significantly differed from each other; Kolmogorov-Smirnov test, *p* = 0.25). Interestingly, NIf_HVC_ spike patterns during this quasi-repeat (two transparent black boxes) also forms a quasi-repeat, with clear differences visible in one of the spike bursts that was composed of 3.3 spikes on average for the first rendition (left blue arrow) and 1.3 spikes on average for the second rendition (right blue arrow). **B.** We computed syllable onsets and offsets as the time points at which sound amplitudes exceeded a threshold of three standard deviations above baseline (silence). **C.** The average cross-covariance function between NIf_HVC_ spike trains and syllable onsets (black) and syllable offsets (red) reveal that NIf_HVC_ cells tend to spike about 21 ms before syllable onsets (black arrow) and to not spike about 21 ms before syllable offsets (red arrow). **D.** Sound amplitudes peak after NIf_HVC_ bursts. The cross covariance function (black) between NIf_HVC_ spike trains and sound amplitudes averaged over *n* = 16 NIf_HVC_ neurons peaks 46 ms after NIf_HVC_ spikes. The broad peak exceeds a significance threshold (dashed lines) of two jackknife standard deviations (dashed lines). Blue and green curves show cross-covariance functions in juveniles and adults, respectively. All data can be downloaded as part of the supporting information files (S1 Data).

Both single and multi-unit activity displayed rhythmic and stereotyped spike patterns during song motifs (Fig 2A). NIf_HVC_ neurons fired high-frequency bursts (range 100–800 Hz) that were time-locked to individual song syllables. To estimate NIf_HVC_ burst precision, we analyzed the onset time jitter of a set of 10 well-isolated bursts (*n* = 6 NIf_HVC_ neurons) relative to syllable onset and found root-mean square (RMS) burst-onset jitters in juvenile birds of 2.92 ± 0.76 ms (std, *n* = 12 NIf_HVC_ neurons). Burst onsets in adults were slightly more precise than in juveniles (RMS burst onset jitter 1.92 ± 0.52 ms [std, *n* = 8 NIf_HVC_ neurons]), though the jitter difference did not reach significance (*p* = 0.06).

We estimated the time lag at which syllable onsets and offsets (Fig 2B) followed NIf_HVC_ spikes by calculating cross-covariance (CC) functions between NIf_HVC_ spikes and syllable onset/offset events (Fig 2C). On average, NIf_HVC_ activity peaked 20 ms before syllable onsets, and it dipped 20 ms before syllable offsets. A clear lead of NIf_HVC_ spikes on song was also evident in CC functions between NIf_HVC_ spike trains and sound amplitudes. Averaged over all 20 cells, sound amplitudes peaked 46 ms after NIf_HVC_ spikes, though individual cells displayed a broad range of peak latencies from 7 to 61 ms (median latency = 42 ms; Fig 2D). The broadly distributed spike-to-sound latencies suggest that NIf_HVC_ cells fire at short but mostly nonzero time lags of each other. A spike-spike cross-correlation analysis of (serially recorded) NIf_HVC_ neurons confirmed this trend: Cross-correlation functions of song-related spike trains peaked at broadly distributed time lags in the range 0 to 300 ms (median lag 70 ms, *n* = 8 NIf_HVC_ neuron pairs in two birds). Thus, despite their common property of leading vocal sound amplitudes, we found no evidence for strongly synchronized activity among NIf_HVC_ neurons.

NIf_HVC_ cells did not only fire spike bursts prior to song and syllable onsets, but also frequently close to the middle of syllables including harmonic stacks, illustrated in Fig 2A. To test the model that NIf_HVC_ spikes tend to cause transient increases in sound amplitudes at a time lag unique to each cell, we computed the sensitivity index (or d′) of spike-triggered sound amplitudes at the preferred time lag for each cell (i.e., the time lag of peak spike-triggered sound amplitude). The sensitivity index measures the separation between the means of the signal (sound amplitudes at preferred time lag) and the noise distribution (amplitudes at spike time), in units of signal and noise standard deviations. Measured d′ values across the population of cells were very small (median d′ = 0.15, mean d′ = 0.29, range 0.04–1.2, *n* = 20 cells), implying that a temporally locked spike-sound model provides a poor account of NIf_HVC_ firing behavior, despite the consistent peak of song amplitudes 50 ms after NIf_HVC_ spikes (we obtained similar conclusions when using Student’s *t* test to compare sound amplitudes at spike and peak times, *p* = 0.12).

### NIf_HVC_ Song-Related Activity Is Pre-Vocal and Does Not Convey Either Actual or Expected Auditory Feedback to HVC

**Fig 3:**
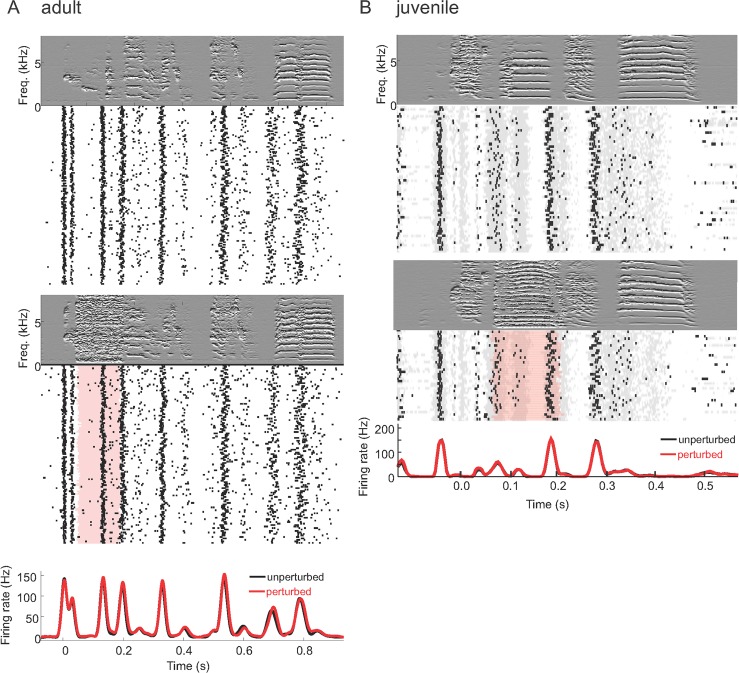
NIf_HVC_ neurons do not signal auditory feedback during song. For both an adult (**A**) and a juvenile bird (**B**), song-related spike raster plots and firing rate curves do not depend on whether the bird was perturbed with a loud stimulus playback during singing (red shaded area, red firing-rate curve) or not (black firing-rate curve). The derivative spectrogram of an example unperturbed trial is shown on top, and of a perturbed trial in the middle. The adult was perturbed with a white noise stimulus, and the juvenile with a long call. The gray shaded area in B depicts raw extracellular voltages in excess of one standard deviation; these are not affected by perturbations, either. All data can be downloaded as part of the supporting information files (S1 Data).

Because songbirds use auditory feedback to learn their songs, it is crucial to understand in which cell populations this feedback is processed and conveyed to motor areas such as HVC. In HVC projection neurons, there are no traces of auditory feedback responses during singing, not even subthreshold [26–28]. One possibility is that NIf is responsive to auditory feedback and that these responses are gated off in NIf-HVC synapses. To test for this possibility, we perturbed birds during song with an auditory stimulus (white noise or zebra finch call) that we time-locked to a randomly chosen syllable using real-time note detection. We produced raster plots of NIf spikes aligned with song motifs. All NIf neurons tested in juveniles and adults (in four adults: *n* = 2 putative NIf interneurons, *n* = 2 NIf_HVC_ neurons, and *n* = 2 multi-unit sites; in four juveniles: *n* = 3 NIf_HVC_ neurons, *n* = 1 putative NIf interneuron, and *n* = 7 multi-unit sites) displayed indistinguishable firing during perturbed and unperturbed songs, even when perturbations were delivered at amplitudes up to 6 dB above maximal song amplitudes (recorded at the microphone roughly 20 cm away from the bird; Fig 3). Given these data, it is very unlikely that NIf neurons in either juveniles or adults convey song-related auditory feedback to HVC.

**Fig 4:**
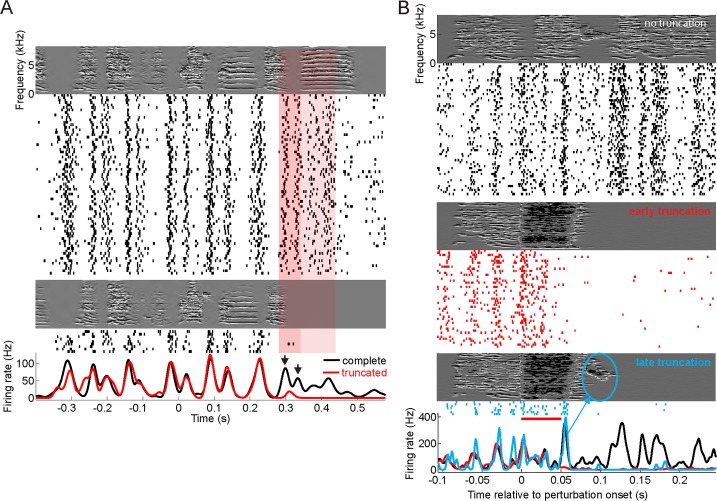
NIf neuronal firing are pre-vocal during song truncations. **A.** Predictive coding by multiple NIf_HVC_ bursts. Spike-raster plot and average firing-rate curves of a NIf_HVC_ cell during complete motifs (top, black firing rate curve) and during nine truncated motifs (bottom, red firing-rate curve), during which the bird dropped the last syllable. Firing-rate curves significantly differed in a large window (red shaded area) starting prior to the last syllable. This NIf_HVC_ cell dropped two bursts (black arrows) during truncated motifs before onset of the last syllable. These bursts were thus associated with the last syllable rather than with the second-to-last syllable. **B.** Raster plots of NIf multi-unit firing and average firing rate curves for truncations that are evoked by a loud 50-ms white noise stimulus (red horizontal bar). Truncations are observed at one of two time points: “early” (red) and “late” (blue). In both cases, termination of NIf activity precedes song offset by about 50 ms. In combination, these truncations allow us to identify the note (blue oval) associated with the last NIf_HVC_ burst. All data can be downloaded as part of the supporting information files (S1 Data).

We found no evidence that NIf neurons signal expected auditory feedback such as could arise, for example, from an efference copy of HVC motor commands relayed to NIf via nucleus avalanche (Av) [29]. Three birds (one adult and two juveniles) truncated their song motifs by occasionally dropping the last syllable. In these birds, during renditions of the non-truncated motif, all neurons and multi-unit sites fired at least one spike within a 50 ms window preceding the onset of the last syllable (*n* = 4 NIf_HVC_ neurons and *n* = 4 multi-unit sites). By contrast, in renditions of truncated motifs, all neurons remained virtually silent in the same 50 ms window (the example cell shown in Fig 4A dropped two bursts in truncated motifs, revealing that behavioral units such as song syllables can be associated with extended sequences of spike trains containing more than a single burst). Based on these truncations, we conclude that NIf activity at the end of the second-to-last syllable was linked to the last syllable but not to expected feedback from the second-to-last syllable. Indeed, in one bird in which perturbations induced the bird to truncate its song, we observed cessation of NIf multi-unit activity, at the earliest, less than 50 ms after perturbation onset (Fig 4B), which is earlier than the median delay of perturbation responses in field L and caudolateral mesopallium (CLM) [5]. The precise stopping time of NIf activity was highly predictive of the ensuing song truncation point, revealing that short-latency NIf firing differences to distorted auditory feedback constitute premotor activity rather than a pure sensory response, because they reflect a sensory-induced change in motor plan. These findings corroborate the notion that rather than being post-vocal (e.g., expected auditory feedback of past vocalizations), NIf_HVC_ activity during singing is pre-vocal (leading vocalizations, possibly including a plan of desired auditory feedback)**.**

### NIf_HVC_ Spike Patterns Preceding Song Notes Are More Stereotyped Than Patterns Following Notes

To assess NIf_HVC_ firing stereotypy relative to brief recurring song notes, we applied a method of birdsong analysis based on efficient coding ideas [30]. For each bird and day of recording, we computed a set of 100 linear filters forming a sparse representation of songs. This representation captured, on average, 94.4% of the song spectrogram variance (range 91% to 98.4% across birds and days). Each filter, when applied to song spectrograms, formed a detector of either a particular song note or variants of a note (see Materials and Methods). When we time-aligned NIf_HVC_ spike patterns with individually detected notes, we found that 80 ms spike patterns preceding notes were very stereotyped; in fact, more stereotyped than patterns following notes (median F_pre_ = 24.3 > median F_post_ = 16.8, sign rank test, *p* < 5 × 10^−5^, *n* = 20 cells). Thus, NIf_HVC_ spike sequences were better aligned with future notes than with past notes. We verified that no filter chosen in the analysis formed a syllable onset detector (S1 Fig), to rule out replication of our previous syllable onset analysis.

NIf_HVC_ firing rates did not correlate with subtle variability of syllables and notes across motif renditions (excluding tempo). In spike raster plots, we selected time windows that contained a single high-frequency burst in most motif renditions and correlated the number of spikes within these windows with the magnitude of the associated note (defined as the maximal filter response within 0 to 80 ms after burst onset). Despite running this analysis for each of the 100 filters and on all 20 NIf_HVC_ cells, we found significant correlations between spike counts and note strength in only 3 of 60 analyzed bursts. The few significant correlations we observed were all seen for sub-notes (a variant of a note that is produced in roughly 50% of motifs; see Materials and Methods and also [30]). Hence, given this predominant lack of correlation between the magnitude of NIf_HVC_ bursts and note strength, we conclude that subtle NIf_HVC_ firing variability is not simply related with subtle variability in produced sound features.

### Local Isometry Between NIf_HVC_ Spike Sequences and Song Notes

NIf_HVC_ spike sequences displayed an isometric relationship with song notes, meaning that preceding similar notes, the spike sequences were similar as well. In some birds, we spotted extended vocal sequences that seemed to repeat. The juvenile song motif shown in Fig 4A contained a quasi-repetition of a sequence of two syllables (“quasi-repetition” because repeated syllables were similar but strictly non-identical; see Materials and Methods). Interestingly, spike sequences in all four NIf_HVC_ neurons recorded during this quasi-repetition also formed a quasi-repetition. Hence, NIf_HVC_ population activity can be similar when preceding similar vocal outputs.

**Fig 5:**
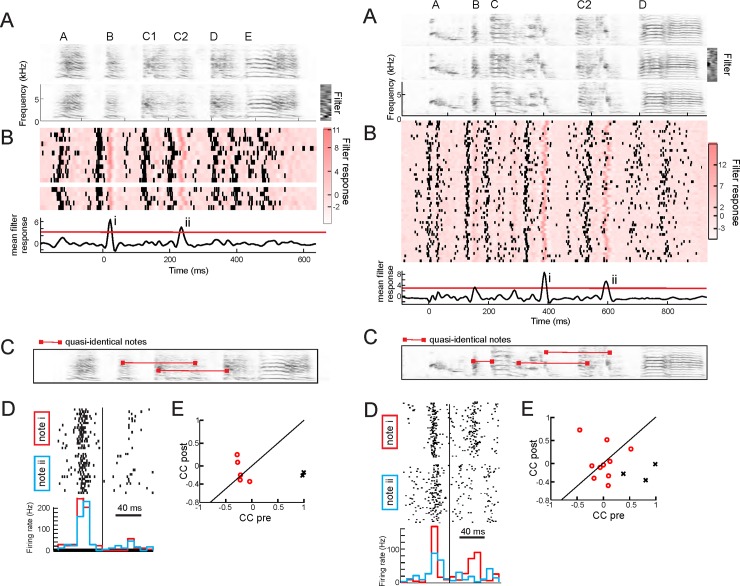
NIf_HVC_ firing patterns are locally isometric with future syllables. Data shown are from two cells in two different birds (left and right). **A.** Spectrograms of three song motif renditions (syllables labeled A–E); black: high sound amplitude, white: low sound amplitude. Right: Example filter. **B.** Spike raster plot superimposed on filter responses, shown as red shaded stack plot. The average filter output (bottom black trace) exceeds a threshold of three (red line) at two locations, defining two quasi-identical notes (marked i and ii). **C.** Quasi-identical notes marked on song spectrogram. The top pair corresponds to the filter in A. **D.** Spike raster plots and binned firing rates (bin size 10 ms) aligned (vertical black line) with individual renditions of each of the two quasi-identical notes. **E:** Scatter plot of correlation coefficients (CCs) of binned firing rates before (pre) and after (post) quasi-identical notes (black crosses). Pre CCs tend to be higher than post CCs, unlike CCs among binned firing rates before (pre) and after (post) non-similar notes (red circles). All data can be downloaded as part of the supporting information files (S1 Data).

**Fig 6:**
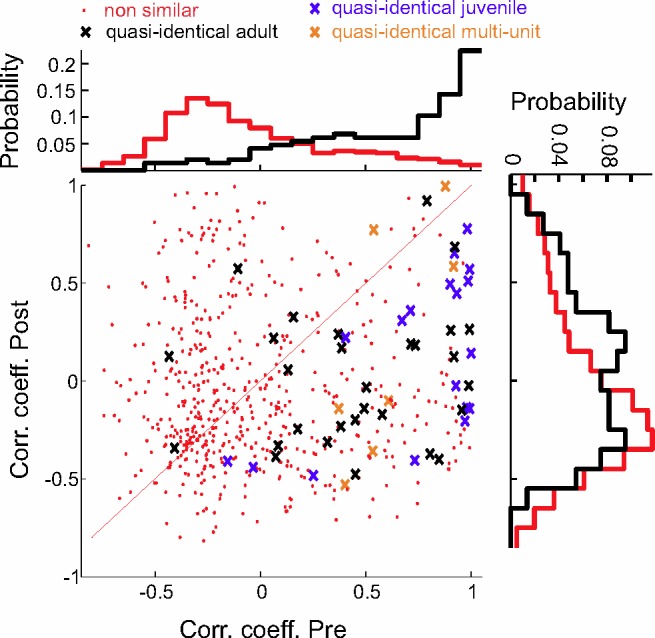
Scatter plot of firing stereotypy before and after quasi-repeated notes. Plotted are CCs of binned firing rates before (pre) and after (post) quasi-identical notes (crosses) versus CCs among binned firing rates before (pre) and after (post) non-similar notes (red dots). Black crosses represent single units in adults, blue crosses single units in juveniles, and orange crosses multi-unit sites. Marginal point densities for single units are shown on top and on the right; they highlight the similarity of spike patterns before similar notes (black, top). Adult and juvenile data are pooled. All data can be downloaded as part of the supporting information files (S1 Data).

To quantify these isometric firing tendencies, we focused our attention on repeated notes. Some birds produced a particular note more than once within single song motifs (*n* = 4 birds). Closer inspection revealed that such repeated notes were highly similar but non-identical (“quasi-identical,” see Materials and Methods). To examine similarity of NIf_HVC_ spike trains preceding a pair of quasi-identical notes, we computed the correlation coefficient among binned NIf_HVC_ spike patterns (*n* = 16 NIf_HVC_ cells) restricted to 80 ms before each note (Fig 5). Correlation coefficients were much higher when the spike patterns preceded quasi-identical notes than when they followed quasi-identical notes (median r_pre_ = 0.71 > median r_post_ = 0.06, *p* < 2 × 10^−7^, *n* = 47 notes, Wilcoxon rank-sum test; Fig 6). Also, spike trains preceding quasi-identical notes were more similar than spike trains preceding non-similar notes (median r_random_ = –0.15, *p* < 2 × 10^−15^, Wilcoxon rank-sum test; Figs 5 and 6). Thus, NIf_HVC_ activity reflected a high similarity of ensuing vocalizations by producing highly similar spike patterns.

On average, firing rates in 80 ms windows were higher preceding notes than after notes (f_pre_ = 37.5 Hz, f_post_ = 30.7 Hz). However, these differences could not explain the higher similarity of NIf_HVC_ firing prior to quasi-identical notes: After randomly removing spikes in pre-note windows to equalize average firing rates in pre- and post-note windows, the similarity among NIf_HVC_ spike trains preceding quasi-identical notes was still exceedingly high (median r_pre_ = 0.66 > median r_post_ = 0.07, *p* < 10^−6^; median r_random_ = –0.15, *p* < 2 × 10^−14^, Wilcoxon rank-sum test).

### NIf Activity Leads Field L Activity during Singing in a Motif-Specific Manner

**Fig 7:**
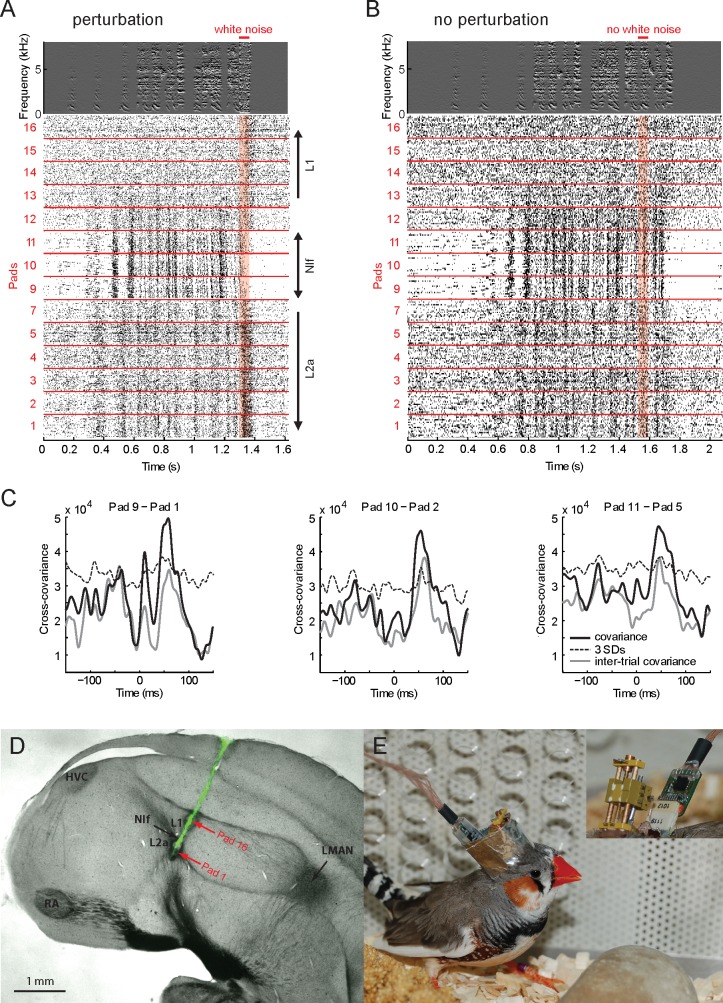
Song-related multiunit activity in NIf leads field L activity. **A.** Distortion of auditory feedback by playback of white noise during song (red horizontal bar) leads to interruption of both song and NIf activity (pads 9–11) but evokes increased responses in fields L1 (pads 13–16) and L2a (pads 1–7). Activity on pads 6 and 8 is not plotted because of excessively high electrode impedance. **B.** NIf, field L1, and L2a activity during unperturbed singing (same animal and electrode array position). The part of the song in which noise is played in A is marked “no white noise.” **C.** Cross-covariance function of simultaneously recorded neuronal activity of three example pairs of recordings in field L2a and NIf, averaged over song renditions (full black line; the dashed line shows 3 standard deviations). The CC function exceeds a shuffle predictor (grey line) of covariance between NIf activity during one song and field L2a activity during the following song (averaged over songs). **D.** Histological verification of recording location. The electrode track is marked with a fluorescent dye (Dil). Red arrows indicate locations of electrolytic lesions that were made on electrode pads 1 and 16. Most pads were in the field L—NIf complex with the first pad potentially located in the caudal auditory striatum (CSt) below field L2a. **E.** A zebra finch carrying a 16-channel silicon probe mounted on a manual microdrive and connected to a headstage. The inset shows the microdrive and the digital headstage without shielding. All data can be downloaded as part of the supporting information files (S1 Data).

We hypothesized that there is a simple predictive relationship between NIf’s motor code and the neural representation of auditory feedback in field L. To directly compare NIf’s motor code with field L’s feedback responses, we recorded multi-unit activity during singing simultaneously in NIf and in the surrounding lateral field L using a 16-pad linear electrode array (silicon probe, see Materials and Methods). Electrodes were implanted such that the tip of the electrode array was located in field L2a, the middle in NIf, and the top recording pads picked up signals from field L1 (Fig 7). Multi-unit firing during singing increased markedly in L2a and in NIf but only weakly in L1 (Fig 7A and 7B). Playback of a loud white-noise stimulus during singing (song perturbation) caused the bird to truncate its song; but unlike in NIf, neurons in field L1 and L2a responded strongly to such white-noise stimuli, thus demonstrating that in field L we recorded feedback-sensitive neurons [5].

A CC analysis between NIf and L2a activity during unperturbed singing showed that NIf firing did consistently lead L2a responses by several tens of milliseconds. NIf-L2a CC functions peaked significantly (more than three standard deviations above zero correlation, *p* < 0.01) at lags within the range 42–58 ms after NIf spikes (*n* = 33 pad pairs in NIf and L2a from 36 possible pairs, *n* = 3 birds, Fig 7C). Additionally, the CC at a 50-ms lag was significant in 28/36 NIf-L2a pairings examined. Interestingly, in all cases, the CC peaks also exceeded by, on average, 18% a shuffle predictor defined by cross-correlating NIf activity during song rendition *i* with field L2a activity during song rendition *i* + 1 (circular boundary condition). Hence, small variability in NIf firing was followed by aligned variability in field L2a, suggesting that NIf firing encodes a motor plan in a representation that is matched by sound sensitivity in field L2a. To inspect the fraction of field L’s song-related response variance predicted by NIf activity, we modeled the mean-subtracted firing rate *f_L_*(*t*) in field L2a as a constant times the mean-subtracted firing rate *f_N_*(*t*) in NIf 50 ms earlier: *f_L_*(*t*) = *cf_N_*(*t* − 50 ms), with *c* being a multiplicative constant we determined for each recording site on training data of paired field L2a–NIf recordings during singing. We found that this simple linear regression model with a single scalar parameter (the constant *c*) accounts, on average, for about 20% of firing variability in field L (on test data excluded during training). Thus, on the coarse level of multi-unit activity, the pre-vocal code in NIf was predictive of a neural representation of auditory feedback.

## Discussion

We showed that NIf firing during song is motor-related and that it encodes neither actual nor expected auditory feedback of past vocalizations. First, distortions of auditory feedback during singing did not elicit differential NIf_HVC_ responses that have been shown to exist in field L [5] and other higher auditory areas [31]. Second, the firing stereotypy of NIf_HVC_ neurons was higher before song notes than after notes, supporting pre-vocal (predictive) rather than post-vocal (postdictive) coding. Third, locally, we found an interesting anticipatory congruence between NIf_HVC_ spikes and vocalizations, manifest as a preservation of similarities between spike patterns and subsequent song features. Namely, in 60–100 ms windows preceding quasi-identical notes, mutual similarity of NIf_HVC_ firing patterns was much higher than expected by chance, even after discounting for biases in mean firing rates. Thus, NIf_HVC_ firing is isometrically linked and strongly constrained by future vocalizations. Fourth, we found NIf_HVC_ activity to lead by several tens of milliseconds auditory activity in surrounding field L. Together, these findings suggest that NIf has a role in motor planning of sensory targets, in alignment with the reduced complexity of Bengalese finch songs following NIf lesions [32].

The vocal lead by 45–50 ms of NIf premotor activity was larger than observed latencies in the downstream motor pathway. For example, the time lag between the robust nucleus of the arcopallium (RA) premotor activity and sound production is on the order of 15–20 ms [33,34]. Combined with an antidromic latency from RA to HVC of about 10 ms [13], we obtain an expected latency from HVC to vocal output of roughly 25–30 ms. Indeed, a recent study found a total latency from HVC to motor output of about 29 ms for isolated projecting cells [35], which is considerably shorter than our measured NIf_HVC_ latencies.

The isometric NIf code might be generated locally, or it could rely critically on communication with other sensori-motor areas including HVC, field L, the caudal mesopallium (CM) [16,20] with its nucleus avalanche (Av) [29], the caudomedial nidopallium (NCM) [36], and nucleus Uvaformis (Uva) [37]. Among these areas, we believe Av to be particularly important due its shared role (with NIf) of driving HVC auditory responses [19].

NIf’s pre-vocal coding of auditory features might be very complex. Namely, across NIf_HVC_ populations, we found no simple relationship between spike rates and vocal output: there were no excessive correlations between spike counts in NIf_HVC_ bursts and the feature composition of song notes. One possibility is that NIf does not code for note variants, another is that the NIf note code might not rely on spike counts. In support of this view, recent studies have shown that in a song pre-motor nucleus, the RA, spike rates are less informative of song features than precise spike-timing codes [38–41].

The role of NIf for singing is comparable with the role of RA for calling. NIf fires in anticipatory manners prior to songs and song syllables, but reversible NIf lesions do not eliminate song [23], just like RA that fires prior to calls [42] but is not required for production of some calls [43]. It is possible that these analogies reflect an evolutionary strategy of linking vocal control areas (HVC, respectively, the dorsomedial nucleus, DM) with upstream planning areas that dynamically trigger behavioral modules but that are not required for their execution.

What might be the origin of NIf's local firing isometry? We speculate that a local isometry could be a remnant of a vocal learning strategy based on duplicating an ancestor syllable and individually reshaping its copies into their corresponding targets [44–46]. NIf firing prior to quasi-identical notes may reflect the motor signal of the common ancestor syllable. If true, the NIf code we discovered might constrain the space within which songs can develop by limiting acoustic deviations from ancestor notes.

How does our interpretation of the isometric NIf code relate to motor control concepts? Our findings agree with the notion that NIf acts as a feedforward controller driving motor gestures encoded in HVC and allows birds to incorporate desired acoustic features into their songs [47–49]. In engineering terms, a link from desired auditory features (in NIf) onto corresponding motor commands (in HVC) is known as an inverse model. The synaptic connections from NIf to HVC could constitute a mapping of desired vocal output onto the motor commands causally required to generate that output, implying that an inverse song model could be stored in the synaptic patterns linking NIf with HVC. Possibly, nidopallial vocal-control areas might share a common strategy of computing inverse models [50]. Premotor codes that are informative about expected sensory feedback are abundant. For example, to support visually guided reaching arm movements, neurons in many premotor brain areas of monkeys have eye-centered reach fields; neurons in regions of posterior parietal cortex and in the medial intraparietal area respond when monkeys prepare to reach for a target within the reach field [51–54]. It was argued that one benefit of these eye-centered coordinates is to allow for simple communication of spatial information among parietal lobe areas. For similar reasons, such communication strategies would also be beneficial to vocal control.

## Materials and Methods

### Ethics Statement

All procedures described here were approved by the Veterinary Office of the Canton of Zurich, Switzerland.

### General

Male zebra finches (*Taeniopygia guttata castanotis*), ranging from 28 to 400 days of age, were obtained from our breeding colony or from a local supplier. Adults and juvenile birds used were raised with their parents and siblings. Two to three days before surgery, the birds were placed in a cage inside the recording chamber for song recording. In case of chronic neuronal recordings, birds were kept there for the remainder of the experiment. Except for three neurons in one adult bird, all recordings in freely behaving birds were made in an undirected setting (no other bird present in the chamber).

### Electrophysiology

Electrophysiological signals were amplified, filtered, and sampled at 32 kHz.

#### Single-channel metal electrodes

Single- and multiunit recordings were made with a motorized microdrive holding one to three glass-coated platinum-tungsten electrodes (Thomas Recording, Giessen, Germany) with impedances in the range 4–14 MΩ. NIf was localized and identified using antidromic stimulation in HVC as previously described [49,55]. NIf neurons were classified as either interneurons (NIf_I_) or as HVC-projection neurons (NIf_HVC_) by near threshold stimulation response variability and antidromic spike collisions. We recorded from 14 antidromically identified NIf_HVC_ neurons and from six putative NIf_HVC_ neurons, in which we were unable to perform antidromic identification for technical reasons. In these six neurons, we ascribed neuron type based on known firing properties [55]. In many recordings with identifiable single-unit activity we also simultaneously recorded multiunit activity that we analyzed in terms of RMS voltage trace. In four cases in which single-unit isolation was judged insufficient, we analyzed spike responses using thresholding of raw voltage traces (Figs 3A and 4A).

#### 16-channel probes

In four birds, we successfully recorded multiunit activity in NIf and field L using either 16-pad linear silicon probes H-16 (Neuronexus, Ann Arbor, USA) or custom-made carbon fiber tetrodes [56]. In case of silicon probes, simultaneous recordings were performed in L1, NIf, and L2a using 15 μm thick linear arrays with pad spacing 50 μm, pad surface 413 μm^2^, and impedance 1.23 ± 0.17 MΩ (Mean ± SD, range 0.96–1.61 MΩ); or pad spacing 25 or 50 μm, pad surface 177 μm^2^, and impedance 1.61 ± 0.35 MΩ (Mean ± SD, range 1.04–2.72 MΩ) at 1 kHz. Silicon probes were mounted on a custom-made mechanical microdrive (see Fig 7E). The headstage for acquiring neuronal signals was built on the basis of an analog frontend with 200x amplification (RHA2116, Intan Technologies, Los Angeles, USA) and an analog-to-digital converter (AD7980, Analog Devices, Norwood, USA) following instructions of RHA2116 datasheet. The 16-MHz clocked digital signal from this headstage was routed through a microcontroller (8-bit C8051F364 operating at 128 MHz, 3.6 V, with passive heat sink, Silicon Labs, Austin, USA), converted, and directed to a digital-to-analog converter (16-channel 16-bit AD5360, Analog Devices, Norwood, USA). Analog signals were acquired using a data acquisition card (PCIe-6259, National Instruments). This setup allowed us to use already custom-written data acquisition software. Neuronal signals were recorded in the Voltage range ±6.25 mV (±5 mV with guaranteed linearity) and in the frequency ranges 45–7,500 Hz or 1–7,500 Hz. The frontend RMS noise was about 2 μV, exceeding the converter step of 0.1907 μV. The dimensions of the headstage were 11.0x8.6x3.3 mm, and its weight 0.4 g. Spike sorting of song-related NIf signals was impossible because of high firing density.

The dorso-ventral movement range of the silicon probe was 6 mm. The adjustment was done using a manually operated screw advancing the probe carrier by 250 μm per revolution. In addition, to allow multiple brain penetrations, the probe could be moved in a range of ±0.5 mm laterally by sliding the probe carrier along two sprigs. The lateral position of the carrier was adjusted using two horizontal screws pushing the carrier into opposite directions (Fig 7E, inset; one screw is visible between two holes holding the horizontal springs). To move the carrier, one screw had to be retracted and another advanced. Position adjustment in the anterior-posterior direction was done by rotating the *d* = 1 mm electrode holder (metallic pin in Fig 7E) to which the electrode was glued eccentrically on the most proximal side to the head midline. A side screw prevented unattended rotation of this pin, which was made of soft Sn60Pb40 solder alloy to allow adjustment of the electrode orientation after being glued to the pin.

#### Histological verification of electrode location

At the end of each experiment, small electrolytic lesions were made in vicinity of the recording sites, animals were killed by pentobarbital overdose, and the brains were removed for histological examination of unstained slices to confirm location of recording sites. In the case of 16-pad silicon probes, lesions were done at the first and the last pads. Additionally, the silicon probes were covered by the fluorescent dye Dil for visualization of electrode tracks (for details, see [57]). Electrolytic lesions were also done at stimulating electrodes to verify their location.

#### Song analysis

Vocalizations were band-pass filtered in the range 0.3–13 kHz and acquired at 32 kHz.

#### Song spectrograms

We computed mean-subtracted log-power sound spectrograms in the frequency range 0–8 kHz. Sound waveform segments of 16 ms duration were tapered with an 8 ms Hanning window and Fourier transformed. Adjacent segments were offset by 4 ms.

#### Note detectors

To identify and detect recurring song notes, we trained a set of linear filters on song spectrograms using an asymmetric version of independent component analysis [30]. The resulting data-derived filters provide a tool for song analysis that is mathematically equivalent to characterizations of sensory neural responses in terms of linear receptive fields [58]. Filter outputs were normal (zero mean and unit standard deviation), but their distribution had a heavy tail along the positive axis; i.e., the filters infrequently responded very strongly to particular song features, clearly indicating absence or presence of that feature. For each recording day, we trained a set of *n* = 100 filters on the complete set of 32 ms-wide spectrogram segments (of which there are hundreds of thousands each day). By virtue of this training, the filters were unique to each bird, determined solely by song statistics. One advantage of such efficient filters over rigid filters are their exhaustiveness; i.e., the filters jointly encode a chosen fraction of total song variance (in our case over 90%).

#### Motif notes

For each recording site, we determined repeated and non-repeated motif notes. First, we temporally aligned song motifs by threshold crossing of sound amplitude; then, for each filter, we averaged filter responses over motif renditions. We searched averaged filter responses for peaks larger than 2.5 or 3 (standard deviations) and less than 30 or 60 ms wide (Fig 4), defining a motif note. The maximum width criterion guaranteed that motif notes corresponded to sharp events in time such as onsets of harmonic stacks but not to the middle regions of such stacks. Individual notes (within individual motifs) were detected as filter response peaks exceeding a threshold of 4 or 6 (SDs) and located within 20 ms of the corresponding motif note. Note that peaks associated with motif notes tended to be lower than peaks associated with individual notes because of considerable fluctuation in song tempo across motifs, which reduces peak amplitude far away from the motif alignment point. Some tuning of parameters was required in each bird to repeatedly detect motif notes irrespective of the motif alignment point.

#### Quasi-identical notes

Motif notes could appear more than once within a song motif, in which case the corresponding peaks (in motif-averaged response) had to be separated by at least 5 ms (Fig 5). We called such motif notes quasi-identical because in all cases they were similar to each other but not identical: the distribution of correlation coefficients among individual note pairs depended on whether the pairs were associated with different peaks or the same peak (in motif-averaged response), Kolmogorov-Smirnov test, *p* < 0.01. Thus, no bird strictly repeated a syllable or a note in the song motif.

For the isometry analysis, we selected all filters associated with pairs (and occasionally triplets) of quasi-identical motif notes. To avoid redundancy in our analysis we only selected filters defining motif notes that were at least 50 ms apart from already selected notes (to disambiguate the filter selection process we first rank-ordered the filters by their smallest motif-averaged peak exceeding 3). The scatter plot of NIf_HVC_ firing stereotypy before and after quasi-repeated notes (Fig 6) depended subtly but not critically on the chosen parameters, such as window size and the initial filters (the filter computation is a non-convex optimization problem).

#### Sub-notes

Occasionally, filters were tuned to sub-notes such as high- or low-pitched versions of a harmonic stack; see also [30]. To exclude such sub-notes from our isometry analysis, we introduced the additional filter selection criterion that the coefficient of variation (CV) of individual response peaks had to be less than 0.2 (the CV is defined as the standard deviation divided by the mean). Using this criterion, individual (quasi-identical or single) notes were guaranteed to occur in nearly each rendition of the song motif.

Note that birds, especially juveniles, differed strongly in variability of song tempo as well as syllable repertoire size. For this reason, we had to fine tune filter-selection parameters in birds until visually satisfying note detection was achieved across days. The parameters we fine-tuned were the response threshold for motif-note detection and the CV criterion for note versus sub-note distinction. Despite such fine tuning, in two birds, no quasi-identical notes could be detected within their song motifs (these birds produced no data for the isometry analysis). The isometry analysis was performed on a total of *n* = 16 NIf_HVC_ neurons.

### Electrophysiological Data Analysis

#### RMS voltage trace

Multiunit activity concurrent with single-unit signal was assessed by the root mean square (RMS) voltage trace *m(t)* in 4 ms moving windows (corresponding to 128 samples each). This RMS value was then thresholded to yield a binary function of time. Separate thresholds were chosen for each recording site.

#### Raster plots

Song-related spike trains were aligned by threshold crossing of the sound amplitude associated with one of the first syllables in the motif. The amplitude threshold was fixed for each bird and set just above noise level during silence periods.

#### Firing rates

To calculate average firing rates, we summed spikes in 4 ms bins and averaged over all aligned motifs. This representation of firing rate is robust to misalignment due to variability in song tempo but does not well capture the high instantaneous firing rates during bursts.

#### Cross covariance functions

To assess the temporal relationship between two time-dependent (and mean-subtracted) functions *ρ*_1_(*t*) and *ρ*_2_(*t*) (e.g., spike trains), we inspected the cross-covariance function

$$C_{12}(t)=\frac{1}{T}\int_{0}^{T}\rho_{1}(\tau )\cdot \rho_{2}(t+\tau )d\tau$$

that reflects the joint spiking density as a function of time lag *t* (Fig 7C). In Fig 2C and 2D we correlated spike trains with sound amplitudes and with syllable on/offset trains (a spike at each syllable onset or offset). Displayed cross covariance functions were convolved with a 4 ms wide Gaussian kernel.

#### Burst-onset jitter

To measure the time jitter of spike bursts, we temporally aligned the spike trains to the closest syllable onset that followed the burst. The jitter corresponded to the RMS time of the first spike in the burst.

#### Auditory feedback manipulations

The method used to assess significance of perturbation responses during manipulations of auditory feedback have been described previously [5]. Briefly, spikes trains were analyzed in 30 ms windows with 25 ms overlap. For a given window, significant differences in the median number of spikes in perturbed and unperturbed trials were assessed using the Wilcoxon rank sum test (*p* < 0.05). Perturbation responses were only counted as significant if at least two adjacent windows satisfied *p* < 0.05.

#### Note-aligned firing stereotypy

We assessed the stereotypy of spike patterns before and after notes using the F-test statistics. For all notes, we produced spike raster plots aligned with individual note time (Fig 5D). We segmented raster plots into 10 ms bins and computed the F value as the variance of average spike counts across bins divided by the average variance of spike counts within bins. For completely random spike patterns, F equals unity, whereas for perfectly stereotyped patterns (determined solely by the average spike count in each bin), F equals infinity. For each cell and motif note, firing stereotypy was evaluated for spike patterns both before and after the note.

#### Firing patterns associated with quasi-identical notes

We computed the similarity of spike patterns in 80 ms windows before quasi-identical notes by cross-correlating note-locked firing rate curves across 10 ms bins (Pearson's correlation coefficient). We similarly compared the similarity of spike patterns after quasi-identical notes. To assess the significance of measured correlations, we also computed a shuffle predictor by randomly pairing individual notes (that were neither identical nor quasi-identical) and by cross-correlating their note-locked firing rate curves (Figs 5E and 6).

## Supporting Information

S1 Fig Linear filters are not syllable onset detectors. No linear filter responded indiscriminately to syllable onsets, presumably because such response would not be sparse enough (100 filters of 32 ms each can cover more than 3s of song material, which is longer than typical zebra finches’ song repertoires). Shown is the response stack (red shading) of the sparse filter that was the closest to a syllable onset detector. The filter detects the onsets of three out of six syllable in this bird’s motifs. The detected syllables start with a broadband note with common low pitch.
(TIF)

S1 Data Archived Matlab scripts and data to plot charts of the article figures. (ZIP)

S1 Text Text file describing the contents of the Matlab scripts. (RTF)

## Abbreviations

Av: nucleus avalanche
CC: cross-covariance
CLM: caudolateral mesopallium
CM: caudal mesopallium
NCM: caudomedial nidopallium
NIf: nucleus interface of the nidopallium
RA: robust nucleus of the arcopallium
RMS: root-mean square; Uva, nucleus Uvaformis
