# Supplementary figures and images for: A Neural Code That Is Isometric to Vocal Output and Correlates with Its Sensory Consequences
Source: PLoS Biol. 2016 Oct 10;14(10):e2000317. doi: 10.1371/journal.pbio.2000317 (PMC5056755; doi:10.1371/journal.pbio.2000317)

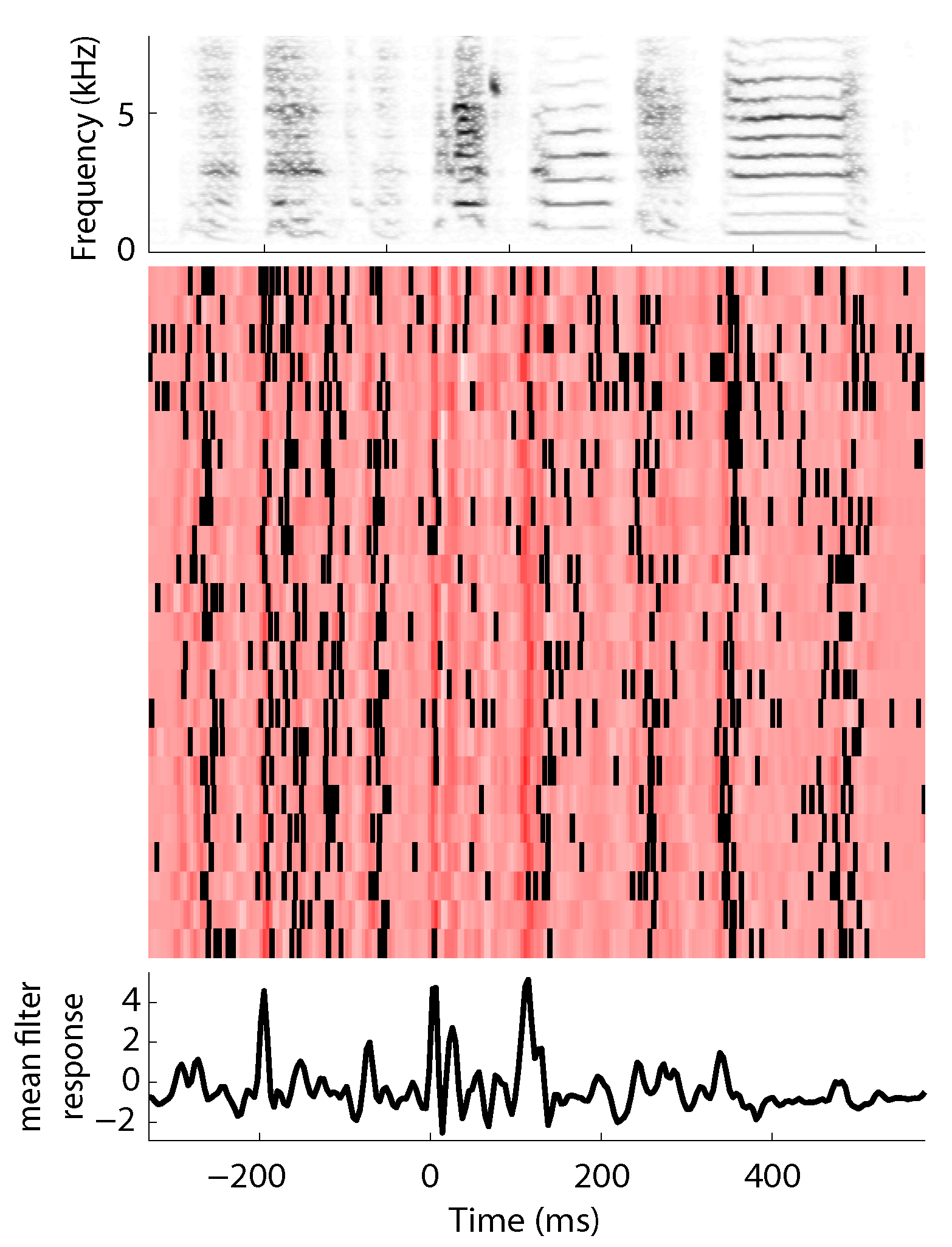

Supplement: S1 Fig — No linear filter responded indiscriminately to syllable onsets, presumably because such response would not be sparse enough (100 filters of 32 ms each can cover more than 3s of song material, which is longer than typical zebra finches’ song repertoires). Shown is the response stack (red shading) of the sparse filter that was the closest to a syllable onset detector. The filter detects the onsets of three out of six syllable in this bird’s motifs. The detected syllables start with a broadband note with common low pitch. (TIF) [file pbio.2000317.s001.tif]
