# Supplementary material for: A Neural Code That Is Isometric to Vocal Output and Correlates with Its Sensory Consequences
Source: PLoS Biol. 2016 Oct 10;14(10):e2000317. doi: 10.1371/journal.pbio.2000317 (PMC5056755; doi:10.1371/journal.pbio.2000317)
Supplement: S1 Text — (RTF) [file pbio.2000317.s003.rtf]

Matlab scripts for producing Fig 1-7 and filter responses in Vyssotski et al. 2016

fig1 produces the spike raster stack plot of onset and offset responses shown in Fig. 1 and it computes the response average across all cells. The variable 'filter' contains the firing rate curves in individual cells.
fig2 produces the motif-aligned spike raster stack plot in Fig. 2 and computes the averaged spike-sound covariance function for juveniles and adults separately.
fig3 produces the spike-raster plots of unperturbed and perturbed song motifs in Fig. 3.
fig4 produces the song-truncation spike raster plots in Fig. 4
fig5 produces the spike raster plot and the average motif-aligned filter output in Fig. 5A and B and the spike raster plots aligned with quasi-repeated notes in Fig. 5d, and the scatter plot of correlation coefficients of binned firing rates before (pre) and after (post) quasi-identical notes.
fig6 produces the scatter plot of correlation coefficients of binned firing rates before and after quasi repeated notes in Fig. 6. Adults, juveniles, and multi-unit sites are indicated in separate colors.
fig7 produces the multi-unit stack plots in NIf and field L in Fig. 7 and computes the covariance functions between NIf and field L activity, and the jackknife bounds in Fig. 7c.
filters produces the filter stack plots for the various birds, like the example shown in Fig. 3C of Blättler and Hahnloser 2011.
